# Automated Detection and Monitoring of Ground‐Nesting Bee Nests Using Drone Imagery and Deep Learning

**DOI:** 10.1002/ece3.72856

**Published:** 2026-01-12

**Authors:** Philippe Tschanz, Thomas Renggli, Jonas Winizki, Achim Walter, Matthias Albrecht, Thomas Keller

**Affiliations:** ^1^ Agroscope, Agroecology and Environment Zurich Switzerland; ^2^ Department of Environmental Systems Science, ETH Zurich, Institute of Agricultural Sciences Zurich Switzerland; ^3^ Department of Soil and Environment, Swedish University of Agricultural Sciences Uppsala Sweden

**Keywords:** computer vision, deep learning, drone imagery, ground‐nesting bees, pollinator conservation, pollinator monitoring

## Abstract

Bees that nest in the soil in self‐excavated burrows comprise the majority of wild bee species and provide important pollination and soil functions, yet many species are threatened. Conservation efforts for ground‐nesting bees are often hindered by limited knowledge of their nesting habitat requirements, in part because nests are difficult to locate and efficient methods for monitoring nesting sites are lacking. Automated detection and monitoring of soil mounds (tumuli) produced by ground‐nesting bees, indicating nest presence, could provide new insights into bee nesting biology and population dynamics while also providing crucial data to support conservation and management. Image‐based methods, such as the automated acquisition of high‐resolution aerial imagery using drones combined with modern computer vision techniques, offer a promising path toward scalable systems for detecting and monitoring bee nest tumuli across large areas. Here, we evaluate the feasibility of integrating drone‐based image acquisition with deep learning to detect tumuli representing bee nests and to distinguish them from other soil surface deposits, such as earthworm casts. We demonstrate this approach on a 120 m^2^ area of a densely populated nesting aggregation of *Lasioglossum malachurum* on bare soil containing numerous earthworm casts. Our model reliably detected bee nest tumuli, achieving an *F*_1_ score of 0.90 (precision: 0.89, recall: 0.91). Misclassifications mainly arose from atypically shaped tumuli (e.g., new and incomplete or damaged), and from cases where tumuli overlapped, but no earthworm casts were confused for bee nest tumuli. This pilot study represents a step toward more efficient monitoring of ground‐nesting bees and demonstrates the potential of this approach under specific conditions. Future work could evaluate its applicability across additional habitats and species and explore alternative methods, such as image segmentation, which may be better suited for cases with less distinct tumuli or extensive overlap among soil mounds.

## Graphical abstract

We demonstrate the feasibility of integrating drone‐based image acquisition with deep learning to accurately detect soil mounds produced by ground‐nesting bees, indicating nest presence, and reliably distinguish them from other soil surface deposits. This approach has the potential to gain new insights into bee nesting biology and population dynamics, while also providing crucial data to support the conservation and management of this important group of pollinators.

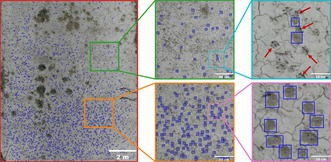

## 1 Introduction

Ground‐nesting bees, that is, bees that nest in the soil in self‐excavated burrows, constitute the majority of wild bee species (Cane and Neff 2011; Danforth et al. 2019; Harmon‐Threatt 2020). They play key roles as pollinators of wild plants (Garibaldi et al. 2013; Michener 2007) and crops (Cane 1997; Kleijn et al. 2015), and also influence various soil functions (Christmann 2022; Tschanz, Albrecht, and Keller 2025). However, many ground‐nesting bees are currently threatened or endangered (Müller and Praz 2024; Nieto et al. 2014), in part due to a lack of suitable nesting resources, disturbances of nesting sites, and accumulation of harmful substances in soils (Harmon‐Threatt 2020; Roulston and Goodell 2011). Despite the importance of nesting habitats for the viability of wild bee populations, our understanding of the nesting habitat requirements of most ground‐nesting bees remains limited due to the difficulty of locating nests and the scarcity of effective methods to identify and monitor nesting sites, which hampers conservation efforts (Antoine and Forrest 2021; Hellerich et al. 2025).

Standardized and efficient detection of ground‐nesting bee nests would simplify the monitoring and collection of conservation‐relevant data of this important group of pollinators. It could also provide new insights into the nesting behavior and dynamics of ground‐nesting bees. Automated image‐based methods for detecting and monitoring the characteristic soil mounds (tumuli) excavated by bees that indicate the presence of a nest, such as the automated acquisition of high‐resolution aerial imagery using drones combined with advanced computer vision approaches, provide a promising framework that can be applied over large areas. However, to our knowledge, the potential of this approach has not yet been assessed.

Aerial imagery from drones and satellites has been successfully utilized to manually detect large soil mounds (0.5 to > 10 m in diameter) made by insects (reviewed in Rhodes et al. 2022), such as ants (e.g., Vogt 2004) and termites (e.g., Mujinya et al. 2014), and efforts are being made to automate nest detection using image segmentation and machine learning approaches (Rhodes et al. 2022). Detecting bee nest tumuli is challenging because these soil mounds are small and less conspicuous compared to those of the ant and termite species studied, typically measuring only a few centimeters in diameter and height (info fauna 2025; Westrich 2018). Moreover, bee nest tumuli often co‐occur with earthworm surface casts, which are similar in size (Edwards and Arancon 2022), presenting additional challenges for reliable and automated detection using traditional image analysis methods. However, bee tumuli are created through soil excavation and, in most species, consist of loose accumulations of relatively fine‐grained soil material (Danforth et al. 2019), whereas earthworm casts are produced through egestion, resulting in more cohesive, pellet‐like mounds (Edwards and Arancon 2022). Therefore, recent advances in computer vision techniques should enable accurate detection of bee nest tumuli and differentiation from other surface deposits such as earthworm casts. Deep learning models based on convolutional neural networks (CNNs) have revolutionized object detection and continue to improve with more sophisticated architectures. Faster R‐CNN, a two‐stage detector widely recognized for its effectiveness in detecting objects from images, first generates candidate regions using a region proposal network (RPN) and then refines and classifies them (Ren et al. 2016). Detection performance can be further enhanced by using a powerful backbone such as ResNet‐50 for feature extraction (He et al. 2016). Integration of a feature pyramid network (FPN; Lin et al. 2017) additionally improves detection of small or variably sized objects by combining features across multiple scales.

In this pilot study, we evaluated the feasibility of combining drone‐based image acquisition with deep learning‐based object detection, using an enhanced Faster R‐CNN with an FPN and ResNet‐50 backbone, to automatically identify individual ground‐nesting bee tumuli. We also highlight how such an automated detection and monitoring system allows to gain new insights into the behavior and ecology of ground‐nesting bees, and how it can provide valuable information for conservation management of this important group of pollinators.

## 2 Materials and Methods

### 2.1 Description of the Study Site and Drone‐Based Image Acquisition

To evaluate the applicability of drone‐based image acquisition and deep learning‐based nest detection, we used a nest aggregation of the ground‐nesting sweat bee *Lasioglossum malachurum* (Apoidea: Halictidae) located on the premises of the Swiss Federal Research Institute Agroscope in Zurich, Switzerland (47°25′48″ N/08°31′11″ E) as a model species. The nest aggregation was situated within a 10.5 × 51 m bare soil plot that forms part of a long‐term field experiment on soil recovery following compaction under different soil management regimes, including a bare soil treatment where vegetation is suppressed through herbicide application (see Keller et al. 2017 for details). The soil at the nesting site was classified as silt loam (USDA system; Soil Survey Staff 2022), consisting of 7% sand, 69% silt, 24% clay, and 1.4% soil organic carbon (see site C in Tschanz, Koestel, et al. 2023). Visual observations confirmed that the visible soil mounds were created by bees, and several females leaving nests were captured and identified as *L. malachurum* by a bee taxonomist (see site C in Tschanz, Koestel, et al. 2023).

**FIGURE 1:**
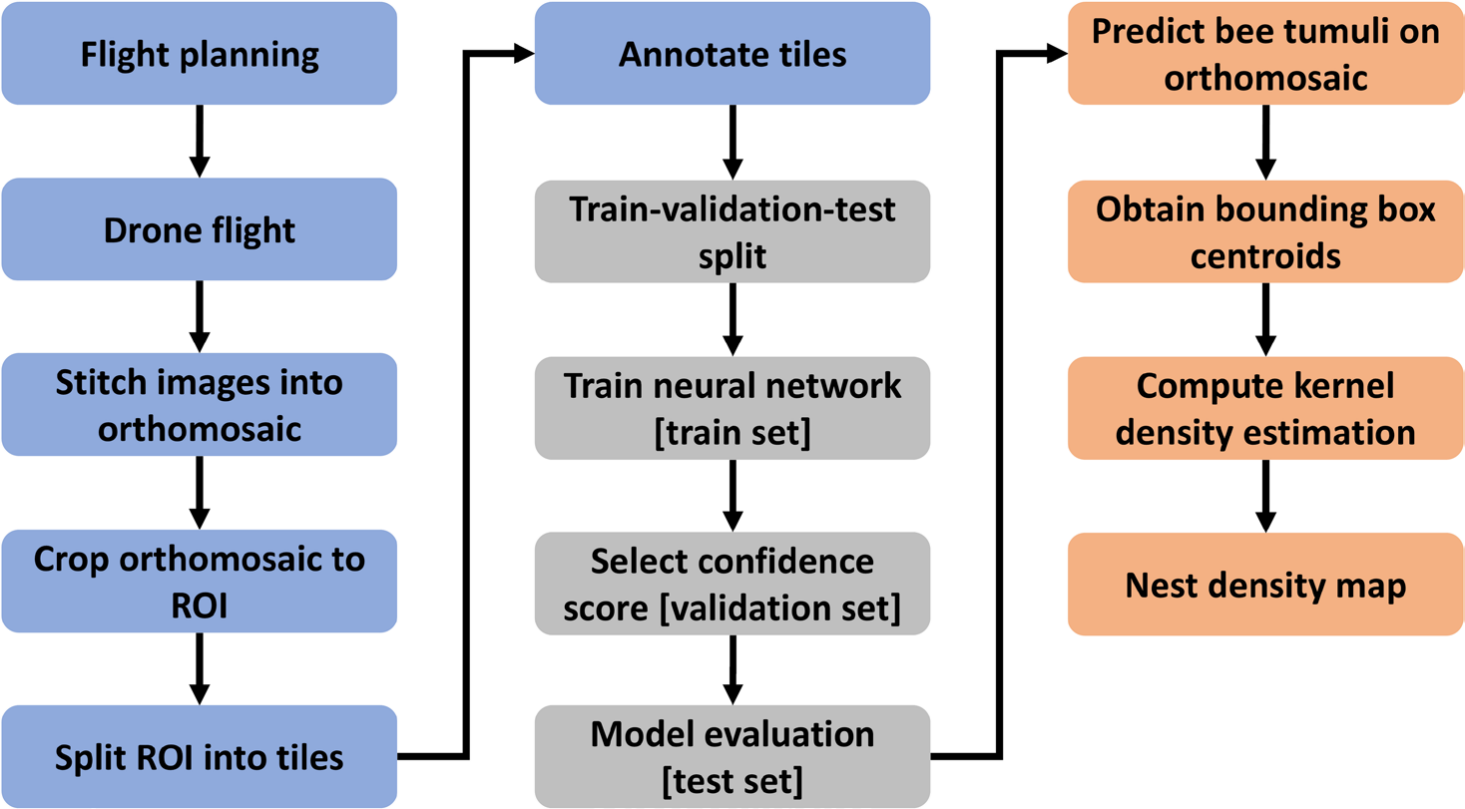
Overview of the workflow used in this study.

The workflow is summarized in Figure 1. Drone images were obtained on April 23, 2020 using an E90 RGB camera (20 megapixel 1″ CMOS sensor) attached to a Yuneec H520 drone (Advanced Technology Labs AG). The flight path was defined in DataPilotPlanner version 1.5.0.1 (Advanced Technology Labs AG) with an image overlap of 85% (both sides) and a flight height of 6.8 m, corresponding to a ground sampling distance of approximately 2 mm per pixel. Before the drone flight, four ground‐control points (GCPs) were installed at the corners of the survey area for geographical alignment. Relative positions and orientation of the collected aerial images were reconstructed and merged into a large orthomosaic using Agisoft Metashape version 1.5.2 (Agisoft, L. L. C 2019). The orthomosaic was cropped to a 10 × 12 m region of interest (ROI) containing a high density of bee nest tumuli.

### 2.2 Model Training

To prepare the dataset for training the tumuli detection model, the orthomosaic (cropped to the ROI) was split into nonoverlapping tiles of 300 × 300 pixels for annotation. All clearly distinguishable bee nest tumuli within these tiles were manually annotated with bounding boxes using the open‐source Computer Vision Annotation Tool (CVAT; Sekachev et al. 2020), and the annotated dataset was exported in COCO (Common Objects in Context; Lin et al. 2014) format. We used COCO format as it is a widely used JSON‐based format that defines how annotations and metadata are stored and is natively supported by the deep learning framework used in this study. The annotated dataset comprised 271 tiles containing 2111 annotated bee nest tumuli in total.

Model training was conducted using GinJinn2 (Ott and Lautenschlager 2022), a deep learning toolkit based on the Detectron2 framework (Wu et al. 2019). ChatGPT‐4o (OpenAI 2025) was used as an assistant for coding. Commands were run in Google Colab (Bisong 2019) to leverage GPU resources. The annotated dataset was divided into training (70%), validation (15%), and test (15%) sets. A Faster R‐CNN model with a ResNet‐50 backbone and FPN from the Detectron2 model zoo (faster_rcnn_R_50_FPN_1x; Wu et al. 2019) was fine‐tuned for nest tumuli detection. The Faster R‐CNN architecture (Ren et al. 2016) outputs the coordinates of the bounding boxes of recognized objects in an input image. FPN improves the performance of the Faster R‐CNN network in detecting objects across scales and enhances the detection accuracy of small objects (Lin et al. 2017), such as nest tumuli. To enhance the variability of the training set, standard data augmentation techniques (i.e., horizontal/vertical flipping, rotation, and changes in image contrast, saturation, and brightness) were applied to each training image, with each technique having a 25% probability of being applied.

During training, model performance was evaluated on the validation set every 200 iterations, using *AP*_50_ (see below for definition) as the primary metric guiding learning‐rate adjustments and early stopping. When *AP*_50_ did not improve for five consecutive evaluations (i.e., 1000 iterations), training was paused and resumed from the best checkpoint with the learning rate reduced by a factor of 10. The initial learning rate was 0.005, which corresponds to the default learning rate scaled to our per‐GPU batch size of four. After no further improvements in *AP*_50_ were observed across five consecutive evaluations at the reduced learning rate (0.0005), training was terminated and the best‐performing checkpoint at iteration 4600 was selected as the final model.

### 2.3 Model Evaluation

Performance of the final nest tumuli detection model was assessed by comparing the predicted bounding boxes with the annotated bounding boxes on the test set using a combination of standard evaluation metrics, that is, *F*_1_ score, precision, recall, and average precision (AP) from the PASCAL Visual Object Classes Challenge (Everingham et al. 2010). The Intersection over Union (IoU) metric was used to determine whether a predicted bounding box matched a ground truth annotated bounding box. A prediction was considered correct (i.e., a true positive) if the IoU of the predicted bounding box with a ground truth bounding box exceeded a threshold of 0.5. The IoU (Jaccard Index *J*) between two bounding boxes, *A* and *B*, was calculated by dividing the intersection area by the union area:

$$J\left(A,B\right)=\frac{|A\cap B|}{|A\cup B|}$$

A confusion matrix (predicted vs. true values) was constructed to obtain the number of true positives (TP), false positives (FP), and false negatives (FN), which were used to calculate the precision, recall, and *F*_1_ score (the harmonic mean of precision and recall) using the following formulae:

$$\text{Precision}=\frac{\mathrm{TP}}{\mathrm{TP}+\mathrm{FP}}$$

$$\text{Recall}=\frac{\mathrm{TP}}{\mathrm{TP}+\mathrm{FN}}$$

$$F_{1}=2\cdot \frac{\text{Precision}\cdot \text{Recall}}{\text{Precision}+\text{Recall}}$$

AP was computed at an IoU threshold of 0.5 (*AP*_50_), as defined in the PASCAL Visual Object Classes Challenge (Everingham et al. 2010).

To determine the optimal prediction confidence score threshold, the *F*_1_ score was computed across a range of thresholds using the validation set. The threshold that maximized the *F*_1_ score (0.45) was selected, as it provided the best balance between precision and recall. Predictions with confidence scores below this threshold were discarded in model evaluation and visualization. Metric computation and visualization were conducted in Python version 3.11.13 (Python Software Foundation 2025).

### 2.4 Nest Density Map

To generate a nest density map of the aggregation within the ROI, the tumuli prediction model was applied to detect bee nest tumuli across the area. Centroids of predicted bounding boxes were used as point locations for kernel density estimation (KDE), producing a continuous map of nest density (nests per m^2^). The KDE bandwidth was chosen by visually inspecting density maps generated across a range of candidate bandwidths (Silverman 2018). A bandwidth of 0.20 m was selected, as it adequately represented the spatial distribution of nests while preserving local density patterns.

## 3 Results

**FIGURE 2:**
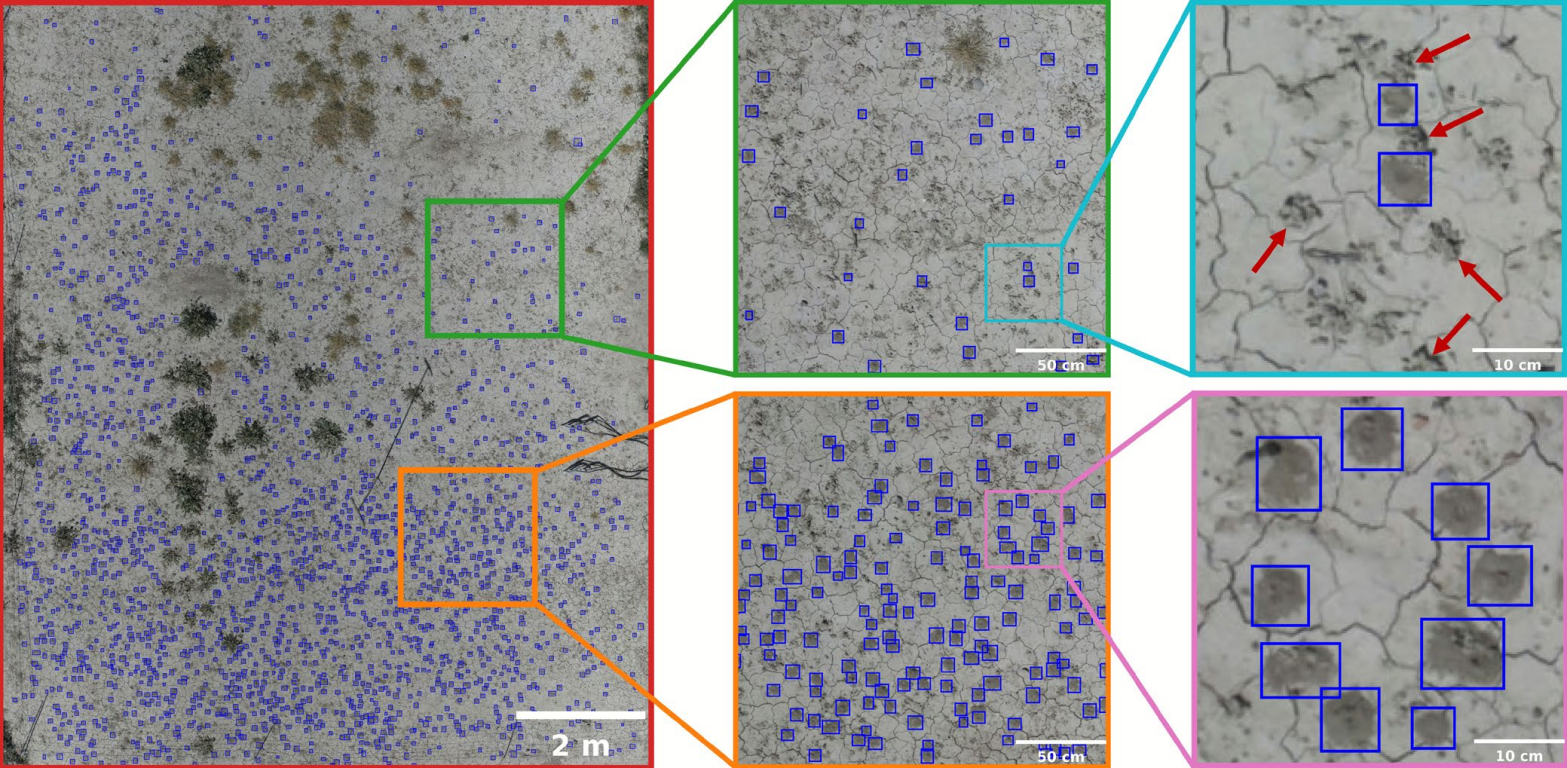
Orthomosaic showing the locations of predicted bee nests with tumuli (mounds of excavated soil material by ground‐nesting bees) delineated by blue bounding boxes at different zoom levels. Red arrows point to examples of earthworm casts that resemble bee nest tumuli in shape and size but correctly distinguished from bee nest tumuli by the trained model.

The trained deep learning model reliably predicted the locations of ground‐nesting bee tumuli (Figure 2). On the test set comprising previously unseen image tiles, the model achieved an *AP*_50_ of 0.93. With an *F*_1_ score of 0.90, a precision of 0.89, and a recall of 0.91, the model effectively located most existing bee nest tumuli while maintaining a low false positive rate (i.e., few misidentifications of earthworm casts or other soil surface deposits as bee nest tumuli).

**FIGURE 3:**
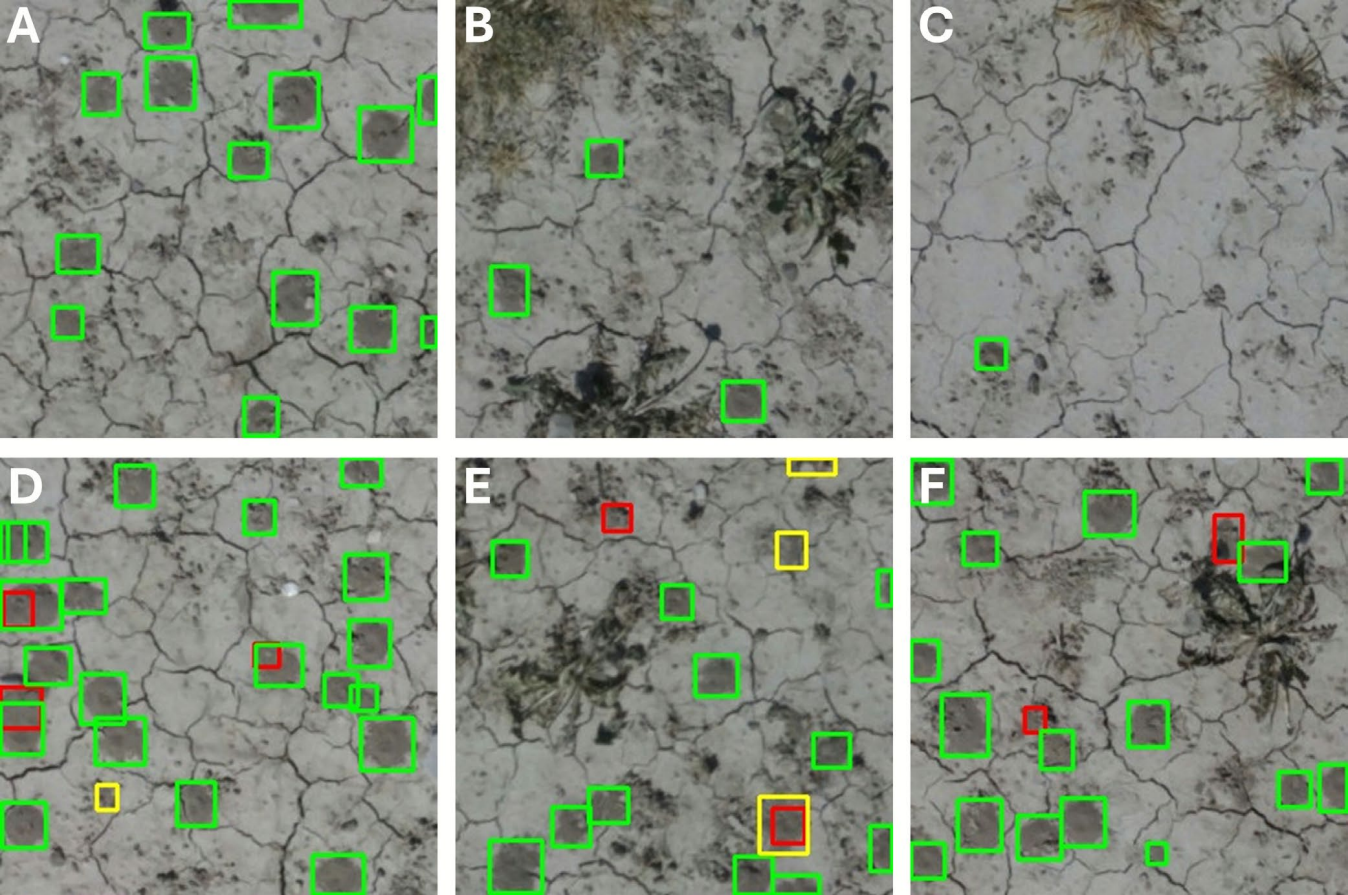
Selection of tiles illustrating the model's performance on the test set without misclassifications (A–C) and typical misclassifications (D–F). Green bounding boxes denote true positives, yellow boxes denote false positives, and red boxes denote false negatives. Typical misclassifications include: partly fused nest tumuli being detected as a single nest tumulus (D); missed detection of annotated nest tumuli due to atypical size and/or shape (e.g., smaller than usual, irregular shape, or fusion of adjacent nest tumuli) (D–F); detection of nest tumuli where no ground truth annotation exists (D, E); inaccurate bounding box size (E); and vegetation partially obstructing nest tumuli (F). False positive predictions also demonstrate the difficulty and subjectivity of classifying a mound as a bee nest tumulus, particularly when the mound is partially destroyed or under construction and thus very small.

Visual inspection of all misclassifications in the test set (33 false positives and 27 false negatives) revealed several likely sources of error. Examples of mispredictions are shown in Figure 3. Most false positives (81%) were potential nest tumuli that were not annotated due to their atypical or inconspicuous appearance (e.g., incomplete and thus very small, irregularly shaped, or disturbed). While we were not sufficiently confident to annotate these structures as nest tumuli, some of these detections may indeed represent true nest tumuli. Higher‐resolution imagery may reduce this error source, although some cases are likely difficult to identify reliably even in the field. Additional false positives resulted from ambiguous soil artifacts (10%), imprecise bounding boxes (6%), and mistaking a stone for a nest tumulus (3%). No earthworm casts were falsely detected as nest tumuli. False negatives arose from several causes. Overlapping tumuli detected as a single tumulus accounted for 30% of missed detections, and partial visibility (e.g., image‐edge cropping, shading, or obstruction by soil features) for 22%. Imprecise bounding boxes contributed to 7% of false negatives. The remaining cases (41%) were likely associated with atypical tumulus morphology or inconspicuous appearance (e.g., small size or low contrast with the background).

**FIGURE 4:**
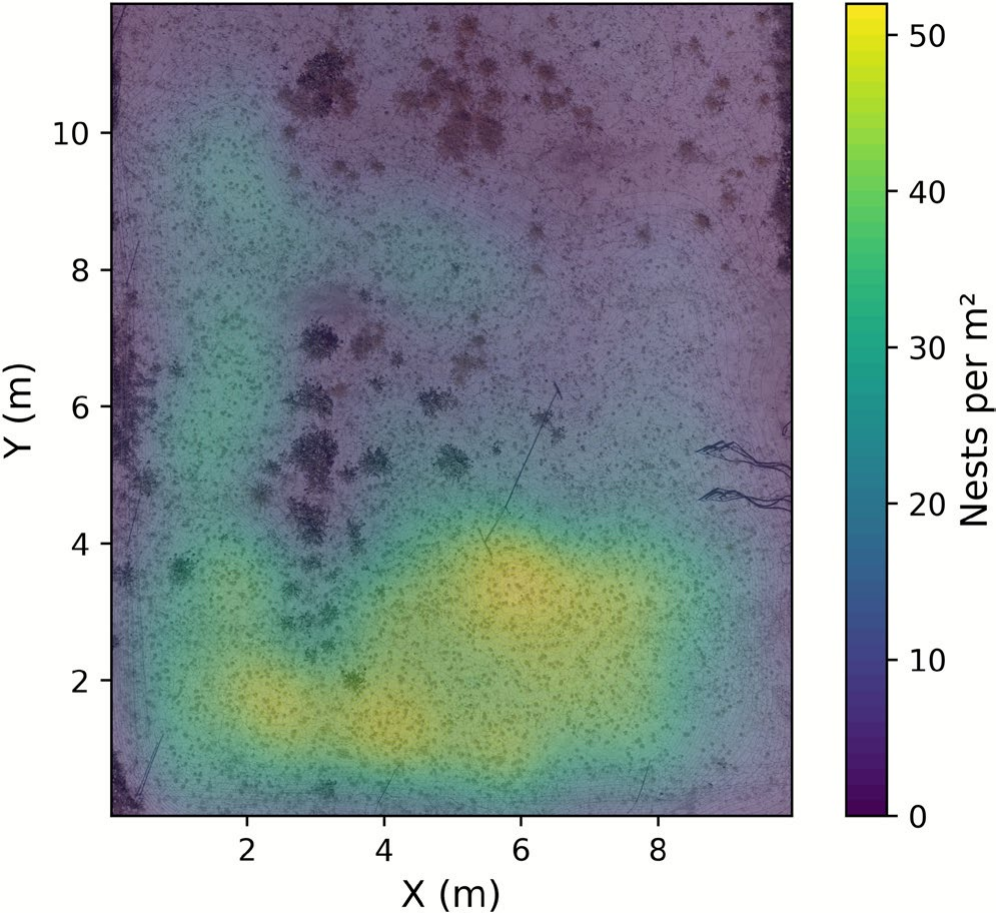
Nest density map of predicted bee nest tumuli derived from kernel density estimation (KDE), showing spatial variation in estimated nest density (nests per m^2^).

Nest density, estimated using KDE with a bandwidth of 0.20 m, varied across the area, with most nests concentrated in the lower (southern) third and a clear avoidance of vegetated patches (Figure 4). The highest nest density was approximately 52 nests per m^2^.

## 4 Discussion

This pilot study demonstrates the potential of an automated pipeline that integrates drone‐based image acquisition with deep learning for detecting soil mounds (tumuli) created by ground‐nesting bees (Figure 2), enabling effective monitoring of bee nests across space and time. The model achieved high performance in both precision and recall, indicating that it was effective at identifying bee nest tumuli while maintaining a low false‐positive rate. Some detection errors did occur, including missed detections, false positives, or where multiple fused tumuli were misclassified as a single tumulus (Figure 3). However, many of these cases were also difficult to reliably identify as a bee nest tumulus through visual inspection in the field, particularly when the tumulus was damaged and had lost its characteristic conical shape, or when the nest excavation had just started and the tumulus was still very small. Despite these challenges, the combined use of drone imagery and deep learning yielded a reliable and objective approach for locating and quantifying ground‐nesting bee nests.

The applicability of our automated approach to nest detection is likely limited to sparsely vegetated areas where bee nest tumuli remain largely unobstructed in aerial imagery. Detecting nest tumuli in densely vegetated landscapes, where vegetation partially or fully obscures the view, may be more challenging or infeasible, but some vegetation may be beneficial for detection because it improves the contrast between tumuli and the vegetated background. Further, the performance and reliability of the approach under other soil and environmental conditions, and potentially altered contrast between bee nest tumuli and the soil surface, or in areas where other soil‐dwelling species produce soil mounds that more closely resemble bee nests than earthworm casts, such as soil mounds produced by ants, beetles, or wasps (Hellerich et al. 2025), remain to be tested. Nevertheless, since many ground‐nesting bee species prefer to nest in sparsely vegetated or bare areas (Antoine and Forrest 2021; Harmon‐Threatt 2020; Westrich 2018), our approach may be applicable across a range of habitats.

An automated pipeline for drone‐based image acquisition and nest detection offers several opportunities to support the conservation and management of ground‐nesting bee populations. It enables noninvasive surveys of large areas to identify nesting sites of conservation concern, estimate population densities of nesting aggregations (Figure 4), and monitor population dynamics over time. Such data are crucial for early detection of population declines, evaluation of habitat improvement efforts, and identification of low‐density nesting areas (Figure 4) to guide targeted habitat improvement measures. This information can also support the management of ground‐nesting bees for crop pollination. For example, the gregarious alkali bee ( *Nomia melanderi* ) is managed in nesting beds for pollinating cultivated alfalfa ( *Medicago sativa* ) (Cane 2024). In these densely populated nesting sites, diseases and parasitoids can spread rapidly among crowded nests (Cane 2024). Early identification of areas with declining populations facilitates investigation of local causes of bee mortality and timely implementation of mitigation measures. Compared with traditional methods, such as visual searches or emergence traps (Hellerich et al. 2025; Klaus et al. 2024), which disturb nesting sites, can be subject to observer bias and may be too labor‐intensive for large‐scale or fine‐grained monitoring, the drone‐based nest detection pipeline offers a more scalable, noninvasive, and objective alternative. Because drone‐based nest detection can survey entire nesting aggregations and does not rely on extrapolation from limited transects or subsamples, this approach may be particularly advantageous for large ground‐nesting bee aggregations, which can exceed 100,000 individual nests (Danforth et al. 2019).

From a more fundamental perspective, an automated nest tumuli detection pipeline could provide valuable insights into the nesting behavior and ecology of ground‐nesting bees. Regular monitoring (e.g., daily drone flights) during the nesting period, coupled with continuous measurements of environmental factors (e.g., air temperature, wind speed, precipitation) and soil conditions (e.g., moisture, temperature, hardness), could deepen our understanding of the nesting biology of ground‐nesting bees. For example, the relationship between bee burrowing and soil mounding activity could be investigated to identify soil hydromechanical constraints on bee burrowing, as has been established for other invertebrates (Ruiz et al. 2023). The temporal dynamics of nesting aggregations could also be monitored across years and related to environmental factors and soil conditions to identify the underlying drivers of population dynamics. Moreover, new insights into the nesting preferences of ground‐nesting bee species could be gained by identifying areas of varying nest densities (e.g., using KDE as shown in Figure 4) and linking these to factors influencing nest site selection, such as soil surface features (e.g., vegetation, landmarks, rocks) and soil properties (e.g., texture, density) and conditions (e.g., moisture, temperature) (Antoine and Forrest 2021; Harmon‐Threatt 2020). A major advantage of the approach presented here is that it enables the acquisition of precise, georeferenced nest locations at daily or even subdaily temporal resolution, which would be infeasible using manual methods. However, because bee tumuli can be eroded by wind, rain, or other physical disturbances, detection accuracy likely declines as tumuli degrade over time. Consequently, the timing of drone imagery is critical to ensure that bee tumuli remain sufficiently intact for reliable detection.

Although our model was trained on tumuli produced by a single ground‐nesting bee species, many other ground‐nesting bee species create morphologically similar tumuli (Tschanz, Vogel, et al. 2023; Westrich 2018) and may also be detected by our model. Nevertheless, expanding the training set to include a broader range of species‐specific tumuli would enhance the general applicability of our approach. For dense nesting aggregations with substantial overlap between bee tumuli (e.g., see figures in Cane 2003; Watanabe 1998), estimating the area covered by bee tumuli using a segmentation approach may be more feasible than detection of single bee tumuli. However, when nest entrances remain clearly visible despite overlapping mounds (e.g., see figure 1 in Watanabe 1998), object detection methods may still perform well.

In conclusion, this study demonstrates the great potential of an automated pipeline that integrates drone‐based image acquisition with deep learning to reliably detect and monitor nests of tumuli‐building ground‐nesting bees in sparsely vegetated areas. It also underscores the potential of such an approach to gain new insights into the biology of ground‐nesting bees and to support the conservation and management of this important group of pollinators. Future research may evaluate the applicability of our approach in more vegetated habitats and for different species of tumuli‐building ground‐nesting bees.

## Author Contributions

**Philippe Tschanz:** conceptualization (lead), data curation (lead), formal analysis (lead), methodology (lead), visualization (lead), writing – original draft (lead), writing – review and editing (lead). **Thomas Renggli:** formal analysis (equal), writing – review and editing (supporting). **Jonas Winizki:** data curation (equal), investigation (lead), writing – review and editing (supporting). **Achim Walter:** supervision (supporting), writing – review and editing (supporting). **Matthias Albrecht:** conceptualization (equal), funding acquisition (equal), supervision (equal), writing – review and editing (equal). **Thomas Keller:** conceptualization (equal), funding acquisition (equal), project administration (equal), supervision (equal), writing – review and editing (equal).

## Funding

This work was supported by Schweizerischer Nationalfonds zur Förderung der Wissenschaftlichen Forschung (185273).

## Conflicts of Interest

The authors declare no conflicts of interest.

## Data Availability

Data and code are available on Zenodo at https://doi.org/10.5281/zenodo.15697085 (Tschanz, Renggli, et al. 2025).
